# Peculiarities of Scattering of Ultrashort Laser Pulses on DNA and RNA Trinucleotides

**DOI:** 10.3390/ijms232315417

**Published:** 2022-12-06

**Authors:** Dmitry Makarov, Anastasia Kharlamova

**Affiliations:** Department of Fundamental and Applied Physics, Northern (Arctic) Federal University, Nab. Severnoi Dviny 17, 163002 Arkhangelsk, Russia

**Keywords:** X-ray scattering, DNA, RNA, X-ray diffraction, ultrashort pulse, trinucleotides, CCG, CGG, CAG, CUG

## Abstract

Currently, X-ray diffraction analysis (XRD) with high spatial and time resolution (TR-XRD) is based on the known theory of X-ray scattering, where the main parameter of USP—its duration—is not taken into account. In the present work, it is shown that, for scattering of attosecond USPs on DNA and RNA trinucleotides, the pulse length is the most important scattering parameter. The diffraction pattern changes considerably in comparison with the previously known scattering theory. The obtained results are extremely important in TR-XRD when using attosecond pulses to study trinucleotides of DNA and RNA, because with the previously known scattering theory, which does not take into account the duration of USP, one cannot correctly interpret, and therefore “decode”, DNA and RNA structures.

## 1. Introduction

It is well known that X-ray diffraction analysis (XRD) is the primary method of determining the structure of matter [1,2,3,4]. The structures of most crystals and complex biomolecules, such as deoxyribonucleic acid (DNA) and ribonucleic acid (RNA), have been determined using this method. The theoretical basis for diffraction analysis dates back to the work of Max von Laue, who won the Nobel Prize in Physics in 1914 for the discovery of how X-rays can diffract on crystals [2]. The diffraction pattern obtained by scattering X-rays from various substances is the “imprint” of this material, whose deciphering enables the determination of crystal structures. The importance of this area can be estimated by the number of Nobel Prizes awarded for work related to X-ray crystallography, of which there are now more than 20. The key quantity in X-ray scattering is the scattering spectrum (determining the diffraction pattern) which is related to the Fourier transform of the electron density distribution ρ (r) in matter as follows [5,6]

(1) $$\begin{array}{c} \frac{d\varepsilon }{d\Omega }=\frac{d\varepsilon_{e}}{d\Omega }{\left|\rho \left(r\right)e^{ipr}d^{3}r\right|}^{2}, \end{array}$$

where $\frac{d\varepsilon_{e}}{d\Omega }$ is the scattering spectrum of a free electron (Thomson scattering), p is the momentum transferred to the electron during light scattering (otherwise, $p=\frac{2\pi }{\lambda }\left(n-n_{0}\right)$ is the scattering vector, where n is the direction of scattered radiation, $n_{0}$ is the direction of the primary radiation), Ω is the solid angle at which scattering occurs. Using known methods [7] it is possible to determine ρ (r) from the scattering pattern of X-rays. The surprising result is that Equation (1) can be derived from the classical description of electromagnetic radiation scattered by stationary electron density ρ (r), which gives a result identical to that obtained with the quantum electrodynamic description of scattering [5]. The simplicity of the Equation (1) has given it wide distribution in XRD using not only conventional X-ray sources, but also sources generating ultrashort X-ray pulses (USPs). Further developments in physics have led to the creation and use of USPs to investigate the structure of matter. This is primarily related to the study of the structure of matter with not only high spatial but also temporal resolution. To study the structure of matter with high temporal resolution, the duration of the X-ray pulse must be shorter than the time scale on which the motion of the electron wave packet unfolds. In this case (e.g., [2,6,8]), instead of Equation (1)

(2) $$\begin{array}{c} \frac{d\varepsilon }{d\Omega }=\frac{d\varepsilon_{e}}{d\Omega }{\left|\rho \left(r,t\right)e^{ipr}d^{3}r\right|}^{2}. \end{array}$$

Equation (2) differs from Equation (1) only by replacing ρ (r)→ρ (r,t). This substitution provides access to the instantaneous electron density ρ (r,t) as a function of time. This method is well known as time-resolved X-ray diffraction (TR-XRD). In other words the TR-XRD technique uses the same Equation (1), but the diffraction pattern is "read off" the system being studied over time *t* and conclusions are drawn about the dynamics of the system from a large set of such patterns.

Although one can see that the Equation (2) does not contain values characterizing USPs, this question deserves a separate consideration. Indeed, particular attention is now being paid to the physics of USPs, namely, the methods for generating such pulses and their interaction with matter [9,10,11]. Using such USPs, one can investigate the structure of complex substances with an advanced structure with high temporal and spatial resolution. Moreover, it is now technically possible to carry out such studies, e.g., using XFELs [12] free-electron lasers. Such lasers can generate USPs not only of femtosecond but also of attosecond duration [13,14]. Despite this, old theoretical approaches for “deciphering” polyatomic system structures based on the use of Equations (1) and (2), which do not take into account the features of interaction of such USPs with complex polyatomic structures are currently used [6,8,15,16,17].

Recently, it has been shown in [18,19,20] that there are important cases where the specific scattering of USPs must be accounted for in diffraction analysis; consequently, the use of Equations (1) and (2) results in significant errors. Basically, such errors appear in the scattering of attosecond USPs on certain kinds of structures. Such structures have been shown to include DNA and RNA nucleotides, etc. Thus, it is important to study structures where previously known diffraction analysis theory would give wrong results. By using such erroneous results when “deciphering” structures, it is possible to incorrectly identify the structures of the object under study. An interesting system to study scattering is DNA and RNA molecules. Let us look at some of the trinucleotides in DNA and RNA: CCG, CGG, CAG, CUG. Trinucleotides or triplets are repetitive regions in a molecule. The arrangement of triplets indicates the presence or absence of DNA and RNA mutations [21]. The mechanism of repeat formation is not fully known, but research in this area is actively pursued [22,23,24,25].

In this paper, it is shown that the USP duration for scattering on DNA and RNA trinucleotides: CCG, CGG, CAG, CUG, is one of the most important characteristics when using attosecond pulses. If the pulses are much longer than attosecond pulses, the duration parameter does not play a significant role in the scattering pattern and one can use Equations (1) or (2). The results show that the use of the Equation (1), on the basis of which complex molecules and biomolecules are “decoded”, should be applied with great caution in the case of attosecond USPs.

Next, we will use the atomic system of units: *ℏ* = 1; |e| = 1; $m_{e}$ = 1, where *ℏ* is the Dirac constant, *e* is the electron charge, $m_{e}$ is the electron mass.

## 2. Results and Discussion

**Figure 1:**
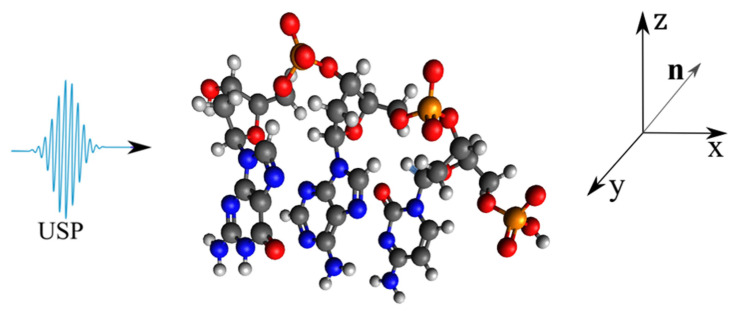
The CAG trinucleotide on which the USP falls is depicted, along with the selected coordinate system. The scattering spectra were calculated in the spatial orientation of the CAG with respect to the USP in the selected coordinate system shown in this figure. The red balls are oxygen atoms (*O*), the yellow ones are phosphorus (*P*), the blue ones are nitrogen (*N*), the light grey ones are hydrogen (*H*) and the dark grey ones are carbon (*C*).

**Figure 2:**
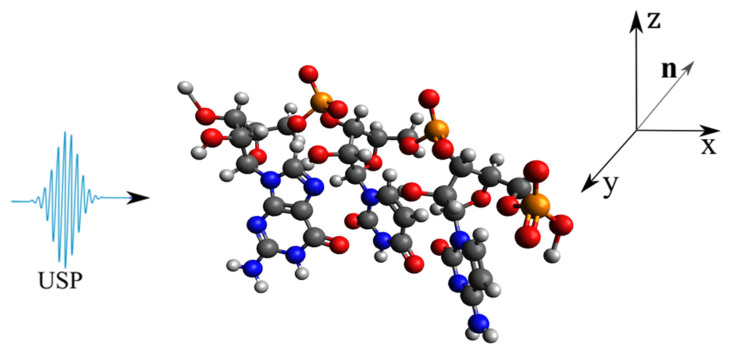
The same as in Figure 1, but only for trinucleide CUG.

**Figure 3:**
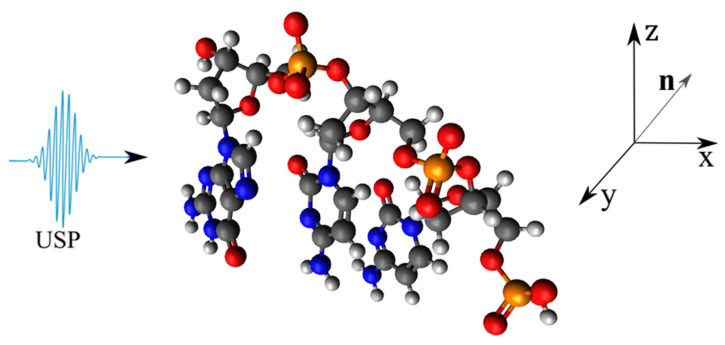
The same as in Figure 1, but only for trinucleide CCG.

**Figure 4:**
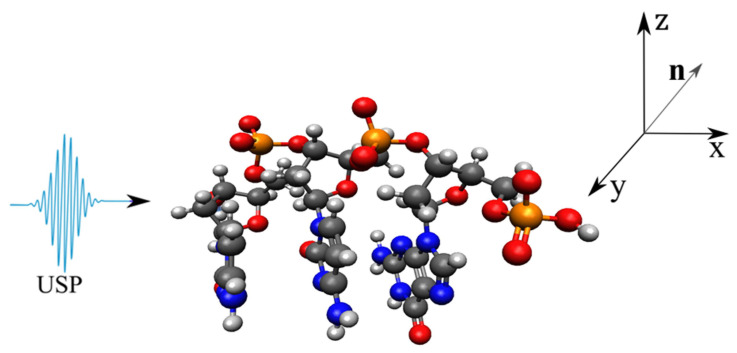
The same as in Figure 1, but only for trinucleide CGG.

As a basis, consider the DNA and RNA trinucleotides: CCG, CGG, CAG, CUG, on which the USP falls in the $n_{0}$ direction, see Figure 1, Figure 2, Figure 3 and Figure 4. Of course, the trinucleotides data presented in Figure 1, Figure 2, Figure 3 and Figure 4 are modeled by us using their famous model. In reality, these trinucleotides can have an altered spatial structure, for example, compressed or stretched, including those without some constituent elements, i.e., atoms. Moreover, we can consider the variant when these structures are destroyed, i.e., when the atoms that make up these trinucleotides move according to some law δR(t)=vt, where *v* is the decay rate, and *t* is the decay time. We will not consider these cases, but will limit ourselves to the trinucleotides presented in Figure 1, Figure 2, Figure 3 and Figure 4. This is entirely justified, since if the duration of the USP will affect the scattering spectra of the trinucleotides shown in Figure 1, Figure 2, Figure 3 and Figure 4, then the length of the USP will play a big role in other cases related to these structures.

We will use the sudden perturbation approximation to find the scattering spectra of USPs. It is assumed that the duration of the ultrashort pulse τ is many times shorter than the characteristic atomic time $\tau_{a}∼1$, i.e., $\tau \ll \tau_{a}$. As has been shown in [26], the condition of applicability of the sudden interaction approximation $\tau \ll \tau_{a}$ need not be fulfilled in the case of X-ray USPs. It has also been shown [26,27] that for X-ray USPs it is sufficient to assume that $\omega_{0}\tau_{a}\gg 1$, where $\omega_{0}$ is the carrier frequency of incident USPs, to extend the sudden perturbation theory to the case of X-ray USPs. In other words, it is sufficient to consider USPs as multi-cycle. We will consider the USP as spatially inhomogeneous, i.e., the USP electromagnetic field strength should be chosen as $E\left(r,t\right)=E_{0}h\left(t-n_{0}r/c\right)$, where $E_{0}$ is the field amplitude, $h(t-n_{0}r/c)$ is the arbitrary function defining the USP shape, *c* is the light speed (in a. u. c≈137). Currently, the USPs intensities at the most powerful XFELs where matter structure studies are carried out do not exceed 10${}^{23}$ W/cm${}^{2}$. In this case (more specifically, at I ≪ 10${}^{25}$ W/cm${}^{2}$), as shown in [18,19,27], the magnetic component of the USP can be neglected. In this case, the scattering spectra (scattered energy USPs per unit solid angle in the unit frequency interval) can be represented as [18,19,27,28]

(3) $$\begin{array}{c} \frac{d^{2}\varepsilon }{d\Omega_{k}d\omega }=\frac{{\left[E_{0}n\right]}^{2}}{{\left(2\pi \right)}^{2}}\frac{|\tilde{h}{\left(\omega \right)|}^{2}}{c^{3}}\left[\sum_{i=1}^{s}N_{e,i}N_{A,i}\left(1-\right|F_{i}\left(p\right){|}^{2})+\sum_{i,j=1}^{s}\delta_{i,j}N_{e,i}N_{e,j}F_{i}\left(p\right)F_{j}^{*}\left(p\right)\right], \end{array}$$

where $N_{A,i}$ is the number of atoms *i* variety; $N_{e,i}$ is the number of electrons in the atom *i* variety; summing over all atoms (i,j) up to *s*; $\tilde{h}\left(\omega \right)=\int_{-\infty }^{+\infty }h\left(\eta \right)e^{i\omega \eta }d\eta$, and $p=\frac{\omega }{c}\left(n-n_{0}\right)$ has the meaning of recoil momentum when a USP is scattered on a bound electron; $F_{i}\left(p\right)=\frac{1}{N_{e,i}}\int \rho_{e,i}\left(r\right)e^{-ipr}d^{3}r$ is the form factor of the *i* atom of the variety with electron density $\rho_{e,i}\left(r\right)$. In Equation (3), factor $\delta_{i,j}=\sum_{Ai,A^{{}^{\prime }}j}e^{-ip(R_{\mathrm{Ai}}-R_{A^{{}^{\prime }}j})}$ is a crucial quantity in USPs scattering, since it depends only on the position of atoms in space, i.e., on the coordinates of the atoms *i* of the variety (with number Ai), whose position in space is determined by the radius vector $R_{Ai}$. In this expression, the coordinates of each of the atoms can be shifted by $\delta R_{Ai}$, thereby simulating various cases from shifts and contractions to decays and synthesis of trinucleotides during TR-XRD, where $\delta R_{Ai}=\delta R_{Ai}\left(t\right)$. In this case, the factor $\delta_{i,j}\left(t\right)=\sum_{Ai,A^{{}^{\prime }}j}e^{-ip(R_{Ai}+\delta R_{Ai}\left(t\right)-R_{A^{{}^{\prime }}j}-\delta R_{A^{{}^{\prime }}j}\left(t\right))}$. It is known that the case of main interest for XRD is at $\tau \omega_{0}\gg 1$, i.e., USP, and is multicycle. If we make the assumption that τ→∞, then we obtain the previously well known XRD theory where the radiation source is continuous. For the calculation, we will use the so-called Gaussian USP, which is the USP where the shape has a Gaussian envelope, i.e., $h\left(t\right)=e^{-i(\omega_{0}t-k_{0}r)}e^{-\alpha^{2}{\left(t-n_{0}r/c\right)}^{2}}$, where α=1/τ, $k_{0}=n_{0}\omega_{0}/c$. It should be added that such a USP is one of the best known for describing the shape of USPs and is often used in theoretical analysis. For example, in [29], based on exact solutions of the electromagnetic field equations, it was shown that at $\omega_{0}/\alpha \gg 1$, the solution takes the form of a simple Gaussian USP. In this case it is not difficult to show that $\tilde{h}\left(\omega \right)=\frac{\sqrt{\pi }}{\alpha }e^{-{\left(\omega -\omega_{0}\right)}^{2}/4\alpha^{2}}$. The condition $\omega_{0}/\alpha \gg 1$ is exactly the same as the multi-cycle USP mainly used in XRD. In this case it is not difficult to obtain the full scattering spectrum using Equation (3) in the form

(4) $$\begin{array}{c} \frac{d\varepsilon }{d\Omega_{k}}=\frac{{\left[E_{0}n\right]}^{2}}{4c^{3}\alpha \sqrt{2\pi }}\left[\sum_{i=1}^{s}N_{e,i}N_{A,i}\left(1-\right|F_{i}\left(p_{0}\right){|}^{2})+\sum_{i,j=1}^{s}\gamma_{i,j}\left(p_{0},p_{\tau }\right)N_{e,i}N_{e,j}F_{i}\left(p_{0}\right)F_{j}^{*}\left(p_{0}\right)\right],\\ \gamma_{i,j}\left(p_{0},p_{\tau }\right)=\sum_{Ai,A^{{}^{\prime }}j}e^{-ip_{0}\left(R_{Ai}-R_{A^{{}^{\prime }}j}\right))}e^{-\frac{1}{2}{\left(p_{\tau }\left(R_{Ai}-R_{A^{{}^{\prime }}j}\right)\right)}^{2}}. \end{array}$$

where $F_{i}\left(p_{0}\right),\delta_{i,j}\left(p_{0}\right)$ at $p_{0}=\frac{\omega_{0}}{c}\left(n-n_{0}\right)$ and $p_{\tau }=\frac{1}{c\tau }\left(n-n_{0}\right)$. The electron density of the atoms $\rho_{e,i}$ variety *i* will be chosen in the independent atom model [30]. In this case, we get $\rho_{e,i}\left(r\right)=\frac{N_{e,i}}{4\pi r}\sum_{k=1}^{3}A_{k,i}\alpha_{k,i}^{2}e^{-\alpha_{k,i}r}$, where $A_{k,i},\alpha_{k,i}$ are constant coefficients defined in [30].

Equation (4) contains characteristics which are responsible for the duration of the τ USP, and hence the specificity of the USP scattering on the system under study is taken into account. In doing so, one can see that if in Equation (4) we increase τ→∞, we get the well known Equation (1) or (2) in case of dependence of atomic coordinates on time, see, e.g., [19]. This is not difficult to show, if τ→∞ with $p_{\tau }=\frac{1}{c\tau }\left(n-n_{0}\right)\to 0$; thus, Equation (4) becomes proportional to τ (Fermi’s golden rule). And Equation (1) in this case, exactly corresponds to the golden Fermi rule. It can be shown qualitatively that the pulse duration τ becomes an important scattering parameter only for attosecond and shorter pulses. For this purpose one can see that in the parameter $\gamma_{i,j}\left(p_{0},p_{\tau }\right)$ in Equation (4) there is an important factor $e^{-\frac{1}{2}{\left(p_{\tau }\left(R_{Ai}-R_{A^{{}^{\prime }}j}\right)\right)}^{2}}$ which is close to unity for small $p_{\tau }\left(R_{Ai}-R_{A^{{}^{\prime }}j}\right)\ll 1$. A small value of $p_{\tau }\left(R_{Ai}-R_{A^{{}^{\prime }}j}\right)\ll 1$ will almost always be the case if the pulses are many times longer than attosecond pulses, since in this case $p_{\tau }≲10^{-4}$. If we choose shorter attosecond pulses, then $p_{\tau }≳10^{-2}$, which leads to considerable values of $p_{\tau }\left(R_{Ai}-R_{A^{{}^{\prime }}j}\right)$ for multi-atomic systems. In this case, the duration of the USP cannot be neglected. Of course, this is a qualitative analysis and one can only say more accurately about the effect of pulse duration after calculations using Equation (4). Although one can quite confidently state that the duration of USPs must be taken into account only for attosecond and shorter pulses. It is also possible to explain qualitatively in terms of physics why the duration of USPs can significantly affect the scattering spectra and why Equation (1) is not always correct. When some fraction of atoms in the matter fall into a spatial region of USP size (∼cτ), the emission is coherent from this region. Accordingly, if the duration of the USP is large, i.e., τ≫1, then the region where emission occurs coherently is also large. In Equation (1), such a region where radiation occurs coherently is infinitely large. This will indeed be true in the case of USP when the region (∼cτ) captures the whole system (molecule, nanosystem, etc.) under study. If, on the contrary, when the ∼cτ region captures a smaller region than the size of the system being studied, there will be no coherent emission from that whole system. That is why the study of USP scattering by bimolecules, especially DNA and RNA, will be relevant, since such molecules are quite large and the size of the attosecond pulse ∼cτ becomes smaller than the size of the molecule itself. This is a very important refinement because when using attosecond pulses in XRD one must take into account the duration of the USP, which is not taken into account in Equation (1). This in turn leads to incorrect determination of the matter structure using Equation (1).

**Figure 5:**
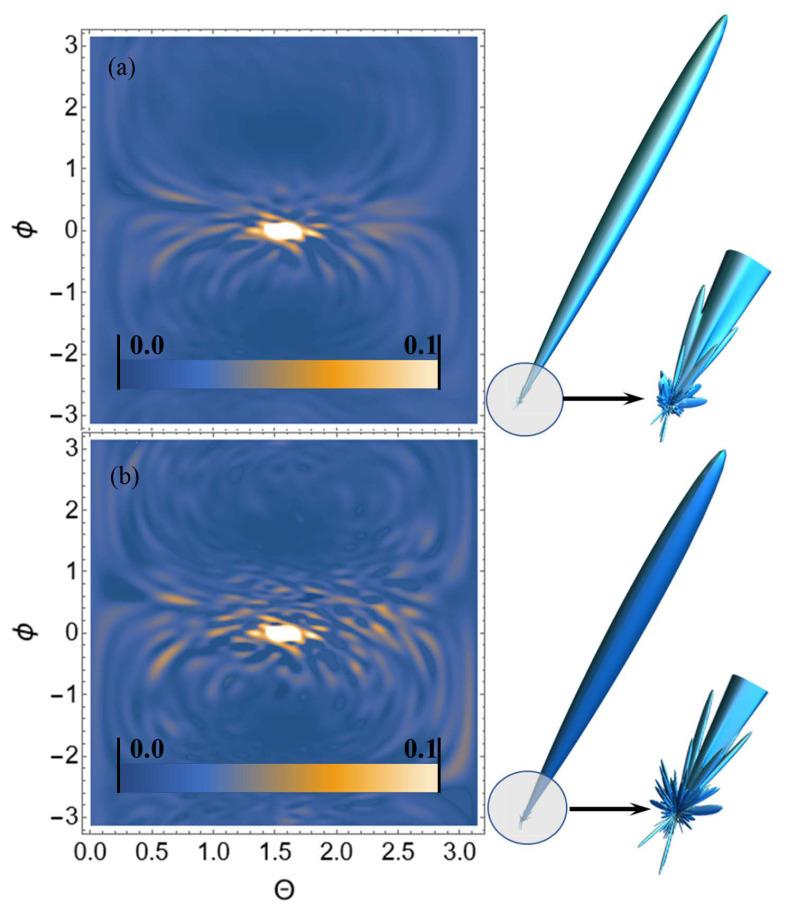
USP scattering spectra on the CAG trinucleotide: (**a**) calculated using Equation (4), (**b**) calculated by Equation (1). The spectra are presented as 2D (**left**) and 3D (**right**) plots. The 2D plots are presented in dimensionless units and normalised to the maximum value of the spectrum. The angles ϕ, θ are spherical angles in the coordinate system shown in Figure 1.

**Figure 6:**
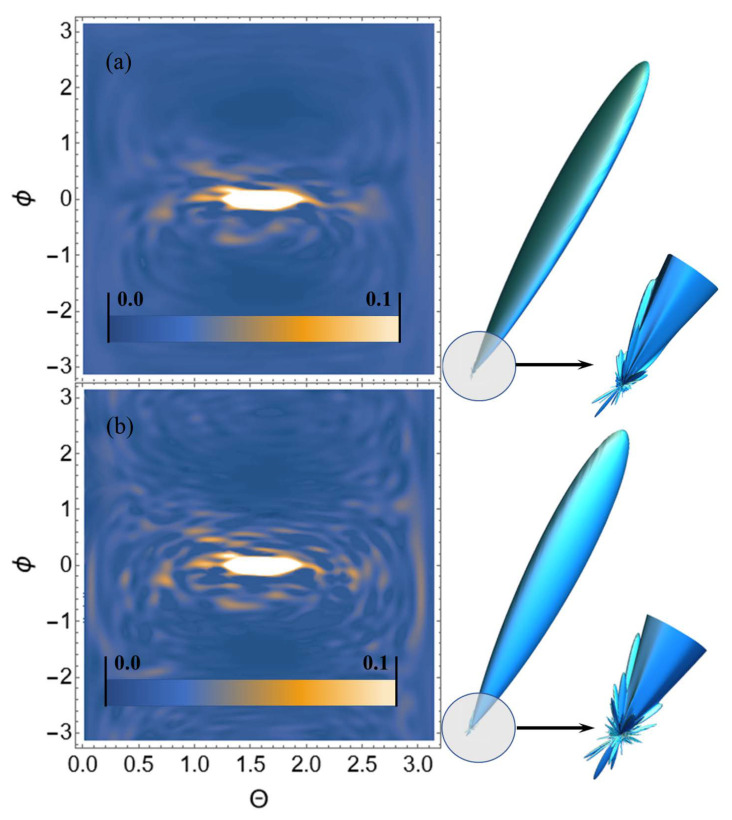
USP scattering spectra on the CUG trinucleotide: (**a**) calculated using Equation (4), (**b**) calculated by Equation (1). The spectra are presented as 2D (**left**) and 3D (**right**) plots. The 2D plots are presented in dimensionless units and normalised to the maximum value of the spectrum. The angles ϕ, θ are spherical angles in the coordinate system shown in Figure 2.

**Figure 7:**
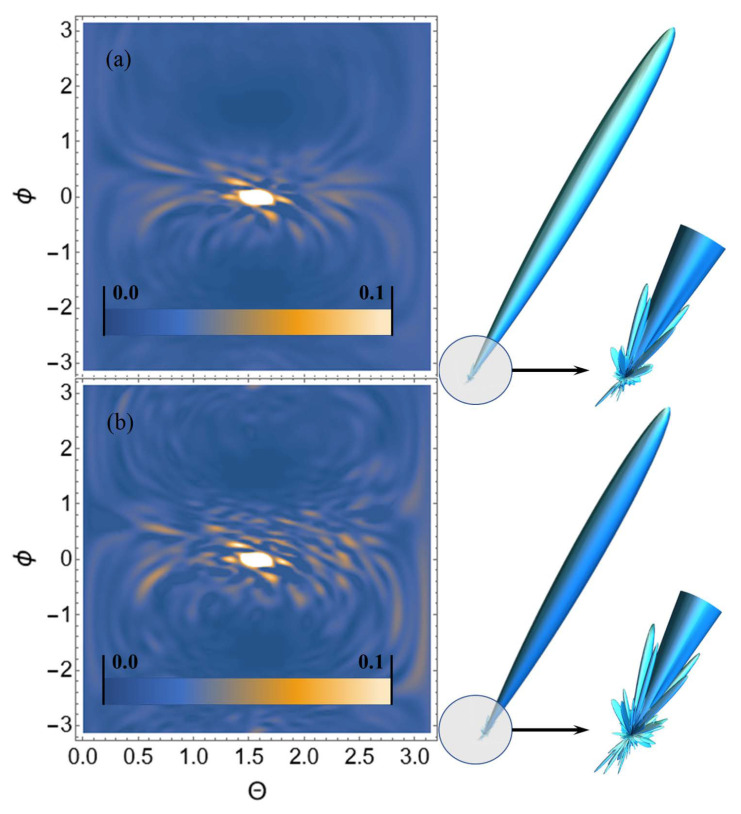
USP scattering spectra on the CCG trinucleotide: (**a**) calculated using Equation (4), (**b**) calculated by Equation (1). The spectra are presented as 2D (**left**) and 3D (**right**) plots. The 2D plots are presented in dimensionless units and normalised to the maximum value of the spectrum. The angles ϕ, θ are spherical angles in the coordinate system shown in Figure 3.

**Figure 8:**
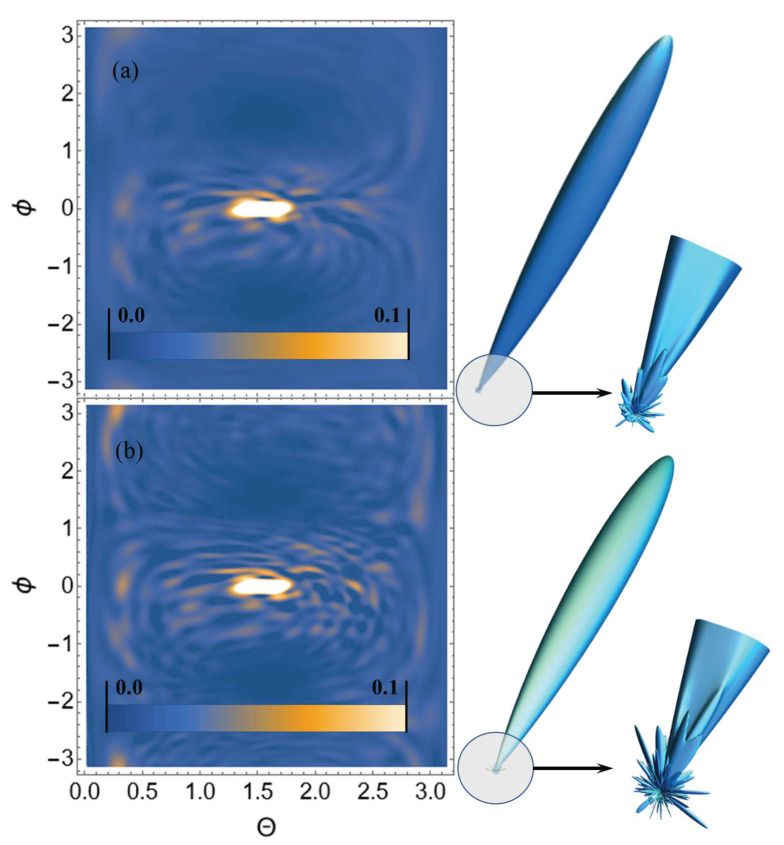
USP scattering spectra on the CGG trinucleotide: (**a**) calculated using Equation (4), (**b**) calculated by Equation (1). The spectra are presented as 2D (**left**) and 3D (**right**) plots. The 2D plots are presented in dimensionless units and normalised to the maximum value of the spectrum. The angles ϕ, θ are spherical angles in the coordinate system shown in Figure 4.

Let us perform USP scattering calculations on the systems shown in Figure 1, Figure 2, Figure 3 and Figure 4 and show that for such systems the use of Equation (1) introduces substantial errors. Calculations of scattering spectra using Equation (4) are shown in Figure 5, Figure 6, Figure 7 and Figure 8. In the calculations, the USP was assumed to fall as shown in Figure 1, Figure 2, Figure 3 and Figure 4 with photon energy $\hbar \omega_{0}=7.46$ keV, pulse duration τ=1 (as). It should be added that the choice of the duration of the USP τ=1 (as) purely conditional, for a better reference in the timeline. If we choose the duration of the tens τ∼10 (as) the results are close, with the only difference being that they are more similar to the results of Equation (1). If we take into account that USPs with a duration of tens of attoseconds [13,14] are already realized, then our theory is feasible in practice.

From the Figure 5, Figure 6, Figure 7 and Figure 8 we can see that the scattering spectra in the case of using Equations (1) and (4) are significantly different. The central peaks, no matter how the calculations were carried out, are always close to each other and this is well explained, since this peak is directed in the direction of the incident USP $n_{0}$. Indeed, it is quite obvious that most of the USP will scatter in the direction $n_{0}$. Other diffraction peaks already differ essentially from each other, and when scattering to more angles they differ even qualitatively, i.e., the scattering is different. At this point, it is possible to notice that the same regularity at scattering, irrespective of investigated trinucleotides, is the reduction of peaks in a diffraction picture at the big angles of scattering in case of using Equation (4). This reduction of peaks in the diffraction pattern can be explained by the influence of the pulse duration on the scattering spectra.

As a result, comparing the results of calculations and analysis of Equation (4), we can draw an important conclusion. In the case of attosecond USPs scattering on DNA and RNA trinucleotides: CCG, CGG, CAG, CUG, the duration of the USPs is an important parameter in scattering, and to use the previously known Equation (1) in XRD or Equation (2) in TR-XRD is not correct. It should be added that this conclusion would have even more significance if we consider that the use of Equation (1) in XRD or Equation (2) in TR-XRD has been standard for many years. Our approach, presented here, for a sufficiently long USP and a multi-atom system, passes to the previously known theory, i.e., Equation (4) becomes equal to Equation (1) in XRD or Equation (2) in TR-XRD. Thus Equation (4) is more general in XRD theory (or TR-XRD), where the previous theory is a special case of the theory presented here. Obviously, in the case of using attosecond pulses, as explained above, to determine structures of more complex molecules than those considered here, the conclusions may be the same. Of course, it is technically quite difficult to study trinucleotides separately, since they are part of DNA and RNA. One way or another, scattering on the basic elements of DNA and RNA (which are trinucleotides periodically repeating in space) gives its own spectrum, and the periodicity from trinucleotides in space in DNA and RNA molecules gives a different spectrum [18,19]. As a result, if the scattering spectra from trinucleotides, using our theory, will differ from the scattering spectra calculated from Equations (1) or (2), then the scattering spectra for the entire DNA or RNA molecule will also differ. Thus, the calculation data obtained and the analysis of Equations (3) and (4) indicate the importance of the conclusions presented here. The main conclusion is the incorrectness of using the previously known XRD or TR-XRD theory in the case of attosecond pulses, where for such pulses it is necessary to use Equations (3) and (4), which take into account the pulse duration parameter. We should also add that here we studied scattering on spatially oriented trinucleotides, which is not the case in the experiment using the pump-probe technique. It is well known that this method has a significant advantage over the standard XRD method [8]. The sample in this method is real, i.e., no additional preparatory procedures are carried out on it, after which the sample may change significantly. It is this method that makes it possible to monitor changes in the structure of complex polyatomic systems in real time. In this technique, the molecules under study are fed chaotically (i.e., random) oriented with respect to the USP. Despite this, it is obvious that if the results in our theory differ from Equations (1) or (2) with a given orientation, then they will also differ with chaotic orientation. This can be clearly seen from Equation (4), where the duration parameter does not disappear when averaged over the orientation of the molecule. Of course, it is quite interesting to give the results of calculations under chaotic orientation of trinucleotides, which we also did, and the results calculated by Equations (1) and (4) are indeed also significantly different as in Figure 1, Figure 2, Figure 3 and Figure 4.

## 3. Materials and Methods

As the methods of our research, we have chosen the theory here developed, see Equations (3) and (4) based on [18,19,26,28]. This method is based on the sudden perturbation approximation [26] and the theory of scattering of ultrashort electromagnetic field pulses [28]. The basic technique for calculating scattering spectra on multiatomic complex systems is presented in [18,19]. Trinucleotides DNA and RNA were chosen as the object of the study: CCG, CGG, CAG, CUG.

## 4. Conclusions

Thus, we can conclude that the theory presented here (see Equation (4)) is needed in TR-XRD to study processes in trinucleotides occurring on an atomic and larger time scale ($\tau_{a}∼100$ as), in the case of using attosecond pulses with a duration of $\tau \ll \tau_{a}$ where Equation (2) gives errors (previously known theory). Also, to study the structure of trinucleotides in XRD, you need to know that when using attosecond pulses, Equation (1) gives errors, and in this case, to determine the structure, it is better and easier to use longer pulses, for example, femtosecond and Equation (1). Although this work is theoretical, it has a very clear practical orientation—it is the deciphering of complex polyatomic structures in both stationary and dynamic states.

## Data Availability

Request to corresponding author of this article.
